# NMDAR inhibitor preconditioned mesenchymal stromal cell-derived extracellular vesicles enhance post-stroke recovery by targeting excitotoxicity and neuronal regeneration

**DOI:** 10.3389/fncel.2025.1608615

**Published:** 2025-08-12

**Authors:** XiaoLu Zhang, HuanNa Tian, HaiMei Bo, Li Zhong

**Affiliations:** ^1^College of Life Sciences, Institute of Life Science and Green Development, Hebei University, Baoding, Hebei, China; ^2^Department of Neurosurgery, The First Affiliated Hospital of Baotou Medical College, Baotou, China; ^3^Hebei Key Laboratory of Nerve Injury and Repair, Institute of Basic Medicine, Chengde Medical University, Chengde, Hebei, China

**Keywords:** extracellular vesicles, ischemic stroke, excitotoxicity, umbilical cord mesenchymal stem cells, preconditioning, neuroprotection

## Abstract

**Background:**

Stroke is a leading global cause of disability and mortality, with ischemic stroke triggering NMDAR overactivation and excitotoxic neuronal injury. Extracellular vesicles (EVs) derived from stem cells under specific microenvironmental conditions show therapeutic potential for stroke recovery.

**Materials and Methods:**

Photothrombotic stroke was induced in male ICR mice, followed by intravenous administration of EVs from memantine-preconditioned human umbilical cord mesenchymal stem cells (HUC-MSCs; M-EV). Behavioral outcomes were assessed using modified neurological severity scores (mNSS) and Morris water maze tests. Tissue damage was evaluated via TTC staining, Evans blue extravasation, and immunofluorescence. PCR-array analysis identified neuronal regeneration pathways. In vitro, oxygen-glucose deprivation (OGD)-challenged HT22 hippocampal neurons were co-cultured with M-EV to assess viability, migration, and apoptosis.

**Results:**

M-EV outperformed conventional EVs in functional recovery, with miR-139-5p and miR-133b identified as key miRNAs enriched in M-EV, mediating neuroprotective effects. M-EV treatment activated neuronal regeneration pathways and reduced infarct volume. In OGD models, M-EV enhanced HT22 neuron viability, promoted migration, and suppressed apoptosis.

**Conclusion:**

Memantine-preconditioned EVs (M-EVs) exhibit superior neurorestorative capacity via miRNA-mediated mechanisms, offering a promising translational approach for stroke therapy. The study highlights the potential of microenvironment-tailored EVs in neural repair.

## Graphical Abstract

MSC-EVs reduce excitotoxicity & promote neurorepair in stroke. 
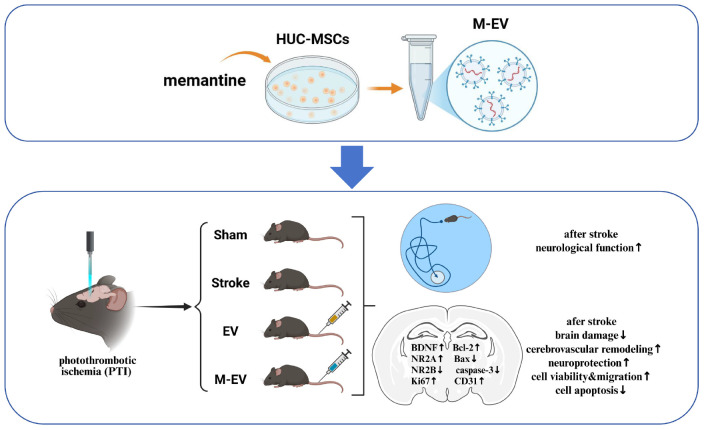

## 1 Introduction

Stroke ranks as the third most common cause of death globally, with 93.8 million survivors and 11.9 million new cases reported in 2021. Worldwide stroke-related mortality reached 7.3 million deaths, representing 10.7% of total fatalities that year (GBD 2019 Stroke Collaborators, 2021). Ischemic stroke is a serious threat to human life and health. Post-stroke functional impairment involves not only physical disabilities but also cognitive impairment, affecting approximately one-third of patients and severely impacting their ability to live independently (Feigin et al., 2014; Kalaria et al., 2016). Ischemic stroke occurs when blockage or occlusion of arteries leading to the brain interrupts the blood supply (Lo et al., 2003). The resulting interruption of glucose and oxygen supply, over-activation of glutamate receptors, calcium overload, acidosis, and oxidative stress lead to rapid neuronal death in the infarcted center (Lo et al., 2003; Moskowitz et al., 2010). Currently, TPA (Tissue-type Plasminogen Activator), an enzymatic agent capable of lysing blood clots, is used to treat acute cerebral ischemia. However, the short treatment window and potential for bleeding limit its clinical application (Powers et al., 2019).

Human umbilical cord mesenchymal stem cells (HUC-MSCs) are widely used in the treatment of stroke due to their immunomodulatory and self-renewal abilities, replacing damaged cells (El Omar et al., 2014; Li et al., 2015). Extracellular vesicles (EVs), measuring 50–120 nm in diameter, are bilayered nanoparticles secreted by diverse cell types, including mesenchymal stem cells, immune cells, and neurons, among others (Yin et al., 2019; Kalluri and LeBleu, 2020; Pegtel and Gould, 2019). These nanoscale carriers demonstrate blood-brain barrier permeability, enabling peripheral-to-brain delivery of neuroprotective signaling molecules. Research reports suggest that EVs can exert similar therapeutic effects as the cells from which they originate. Moreover, UCMSC-EVs can orchestrate multiple regenerative processes after stroke, including neurological functional restoration, cerebrovascular network reconstruction, axonal regeneration, and dynamic remodeling of the neurovascular interface (Zhang et al., 2023; Gregorius et al., 2021; Hou et al., 2020). EVs derived from immune cells enhance disease-fighting immune responses (Veerman et al., 2019), while those from stem cells support tissue protection during regeneration (Newton et al., 2017). Clinically, dendritic cell-derived EVs—containing MHC class I/II antigens and costimulatory molecules have served as immunotherapy agents for non-small cell lung cancer patients undergoing chemotherapy (Besse et al., 2016). This treatment regimen increased natural killer cell antitumor activity. When respiratory syncytial virus infected A549 lung carcinoma cells, their nanoparticle cargo composition changed; administration of these modified EVs stimulated cytokine and chemokine release while activating innate immune responses (Chahar et al., 2018). MSCs pretreated with different microenvironments may exhibit stronger biological activity and therapeutic effects (Li et al., 2019; Ye et al., 2022). Research confirms that miR-126 in serum EVs derived from remote ischemic preconditioning (RIPC) enhances hypoxia tolerance in SH-SY5Y cells through targeted suppression of Dnmt3b, thereby producing neuroprotective effects (Cui et al., 2020). Recent studies demonstrate that ischemia-preconditioned HUC-MSCs-EVs enhance neurovascular restoration and functional neural circuit reorganization in a rat model of ischemic stroke (Ye et al., 2022).

Astrup et al. first introduced the concept of the ischemic penumbra, a region surrounding the ischemic core where neurons remain viable but electrically silent (Astrup et al., 1981). Although this area exhibits neurological impairment, timely restoration of blood flow can reverse dysfunction and improve clinical outcomes. Prolonged ischemia, however, triggers metabolic disturbances that progressively expand the infarct core. Studies demonstrate increasing activation of apoptotic pathways as tissue transitions from the penumbra to the infarct core (Saini et al., 2021). Glutamate receptor overstimulation in this region mediates excitotoxic damage, accelerating both apoptotic processes and neuronal injury. Excitotoxicity drives cell death in acute ischemic stroke, yet pharmacological interventions targeting this mechanism have proven ineffective in clinical settings. Moderate NMDAR activity exhibits neuroprotective properties (Paoletti et al., 2013). However, most NMDAR inhibitors cause severe side effects, including the paradoxical exacerbation of cell death observed at high memantine concentrations. At lower concentrations, memantine effectively shields neurons and glia by disrupting the excitotoxic cascade. These findings support the clinical use of low-dose memantine for stroke-prone patients while cautioning against higher doses (Trotman et al., 2015; Ge et al., 2020). Studies have shown that the composition of EVs are influenced by cellular conditions (Liu et al., 2020a; Panigrahi et al., 2018). Pre-treatment of source cells can simultaneously regulate the compositions of EVs and improve their function. Therefore, we propose using memantine to pretreat HUC-MSCs to produce EVs, in order to achieve efficient treatment of ischemic stroke with low safety risk.

In this study, we use a mouse ischemic stroke model induced by the light clot method to explore the relevant mechanisms of EV-mediated neuroprotective effects pretreated with NMDAR inhibitors. Our results indicate that EVs derived from MSCs pretreated with NMDAR inhibitors promote post-stroke neural regeneration. The up-regulation of miRNAs related to nerve regeneration and the reduction of neuronal apoptosis after OGD/R further emphasize the importance of nerve regeneration in nerve repair. Inhibition of NMDAR-pretreated EVs has been shown to be effective in promoting central nervous system functional recovery after stroke.

## 2 Materials and methods

### 2.1 HUC-MSCs culture and identification

HUC-MSCs are derived from human umbilical cord mesenchymal stem cells that were obtained in our laboratory at an early stage, and there is no ethical dispute. The human umbilical cord mesenchymal stem cells were cultured in serum-free medium (YOKON, China) at 37°C and 5% CO_2_ cell culture incubator (YAMATO, Japan). Perform HUC-MSCs induction differentiation experiments to confirm adipogenesis using Oil Red O staining, validate osteogenic effects using Alizarin Red staining, and detect chondrogenesis using Alcian staining (G1563, Solarbio, Beijing, China). Detect relevant cell surface markers using flow cytometry, including positive markers CD90 (12090942, Invitrogen, CA, United States), CD105 (12105742, Invitrogen, CA, United States), CD73 (550257, BD, CA, United States), and negative markers CD45 (#89492, CST, MA, United States), CD34 (CD3458104, Invitrogen, CA, United States).

### 2.2 EVs extraction and identification

Human umbilical cord mesenchymal stem cells (HUC-MSCs) were cultured in serum-free medium until the cell density reached 90%. The cells were then treated with 200 μM NMDAR inhibitor (HY-B0365A, MCE, NJ, United States) for 24 h. Subsequently, the supernatants were collected and centrifuged at 300 *g* for 20 min. Remove the sediment, then centrifuge the supernatant at 10,000 *g* for 30 min, followed by further centrifugation at 100,000 *g* for 90 min. Discard the supernatant. Resuspend the precipitate in sterile PBS and store at −80°C.

### 2.3 Transmission electron microscopy (TEM)

Observe the morphology and size of EVs using TEM. Apply 10 μL of EVs onto a copper mesh, let it stand at room temperature for 2 min, and use filter paper to remove the excess liquid. Drop a solution of uranyl acetate (and phosphotungstic acid) onto the copper mesh, stain for 1 min, and then use filter paper to remove the excess stain. Allow the mesh to dry at room temperature before observing the samples under TEM.

### 2.4 Nanoparticle tracking analysis (NTA)

Utilize a nanoparticle potentiometric analyzer (Omni, Brookhaven, United States) to measure the size of EVs. Add the test sample to a colorimetric dish for detection. Cover the dish with a lid, then insert it into the sample slot of the analyzer for detection. Record the size distribution of the EVs.

### 2.5 Animal surgery

Sixty male ICR mice (8-week-old, 35–40 g) were procured from SPF Biotechnology Co., Ltd., (Beijing, China) and maintained under specific pathogen-free conditions. All experimental protocols were conducted in accordance with the Guidelines for Animal Experimentation of Baotou Medical College, following approval by the Institutional Animal Care and Use Committee (IACUC Approval No. 2021-012). Photothrombotic ischemia (PTI) modeling was performed as previously described to establish the cerebral ischemic model (Ye et al., 2020). The present study was not pre-registered. Randomization was performed with the online tool QuickCalcs from GraphPad. Rats were coded and assigned randomly to different groups for simple randomization by using: “Random numbers” and “Randomly assign subjects to groups,” in QuickCalcs. 15 mice were randomly assigned to each group. Mice were anesthetized by intraperitoneal injection of 1% Rose Bengal [100 mg/kg dissolved in 0.9% physiological saline] for 20 min. Subsequently, anesthesia was induced by intraperitoneal administration of 2% (w/v) sodium pentobarbital. Fix the mouse on a brain stereotaxic device (Ruiwode, China), the scalp was incised along the midline to expose the skull, followed by periosteal dissection for cranial surface preparation. Stereotaxic alignment was then established by precisely identifying the bregma and lambda landmarks using a calibrated coordinate system Using bregma as the 0 point, determine the stereotactic position on the surface of the skull, with a 1.0 mm opening on the right side and 1.0 mm toward the posterior fontanelle direction (Supplementary Figure 1), and continue to irradiate for 20 min. After completion, suture the scalp and restore normal feeding after anesthesia and awakening. At 24 h post-PTI induction, the mice underwent neuroscore screening (mNSS 11-12) to standardize injury severity. Animals manifesting persistent hemiparesis with preserved brainstem reflexes were stratified into study cohorts, excluding those with confounding neurological complications (Donega et al., 2014). The concentration of EV, as evaluated by the BCA method, was seen to be 0.5 mg/mL. 24 h after PTI induction, EVs were administered via tail vein injection at a dose of 50 μg per mouse (Gupta et al., 2021).

### 2.6 TTC staining

The extent of cerebral infarction was assessed through 2,3,5-triphenyltetrazolium chloride (TTC) staining. Following anesthesia, the mice were euthanized, and their brains were sectioned transversely into five equal parts. Each section was then subjected to a 20 min incubation in a 2% TTC solution (G3005, Solarbio, China), protected from light. Subsequently, the sections were fixed with a 4% paraformaldehyde solution. The area of infarction was determined using Image J software, employing the formula: Percentage of cerebral infarction volume = (Total infarction volume of all slices)/(Total volume of all slices) × 100%.

### 2.7 Modified neurological severity scores (mNSS)

As mentioned earlier, mNSS is used to assess the degree of nerve damage, including monoparesis/hemiparesis, straight walking, beam balancing, and reflex activity (Xiong et al., 2019). At days 1, 3, and 7 after PTI, single-blind measure of neurological function was performed. Higher scores indicate more severe injury.

### 2.8 Morris water maze

The morris water maze (Xmaze, XR-XM101, Shanghai XinRuan Information Technology) experiment were used to detect the neurological function of mouse. Before PTI, all mice underwent a 3-day water maze training period to establish a baseline performance level. Before training, place the platform in the southwest quadrant of the maze. Place the mice, facing the pool wall, into the pool. If a mouse finds the platform, climbs onto it, and stays for 2 s, it is considered a successful trial. If a mouse does not find the platform within 90 s, it is guided there, with their escape latency recorded as 90 s. After the training is completed, induce a PTI model in the mice. Randomly divide the mice into sham, stroke, EV, and M-EV groups and conduct additional training on postoperative days 3, 5, and 7, respectively. On the 7th day, remove the platform and conduct cognitive tests in the form of spatial search experiments with the mice. Record and analyze the escape latency, number of platform crossings, and duration of stay in the target quadrant of the mice.

### 2.9 HE staining

After inducing ischemia with PTI for 7 days, mice were anesthetized by intraperitoneal injection of mixed narcotics. Cardiac perfusion was performed using 4% paraformaldehyde. The brain was carefully dissected, fixed with paraformaldehyde for 24 h, and then dehydrated with a 30% sucrose solution, and sectioned into 4 μm thick slices from peri-infarct zoneon the coronal plane. The sections were placed in hematoxylin for 5 min, differentiated in a differentiation solution for 3 s, and rinsed with running water. They were then dehydrated in 95% alcohol for 1 min and stained with eosin for 15 s. Following this, the sections were dehydrated with alcohol and xylene, and the slides were sealed with neutral gum.

### 2.10 Nissl staining

The sections were placed in Nissl staining solution for 5 min, rinse with running water, differentiate with 0.1% glacial acetic acid, stop the reaction with water washing, dry and seal with neutral gum.

### 2.11 Immunofluorescence staining

The sections were permeabilized with 0.5% Triton X-100 and blocked with 10% normal goat serum. Primary antibodies against CD31 (28083-1-AP, Proteintech, Wuhan, China) and Ki67 (GB111141, Servicebio, Wuhan, China) were applied and incubated overnight at 4°C. Following this, fluorescently labeled Goat Anti-Rabbit IgG H + L(CoraLite594, SA00013-4, Proteintech, Wuhan, China) were added and incubated in the dark for 4 h. The cell nuclei were then counterstained with DAPI (Abcam). The resulting fluorescence intensity was observed, photographed under a fluorescence microscope, and analyzed using Image-J software.

### 2.12 Realtime-PCR

Total RNA was extracted from the cultured cells using TRIzol (DP424, Tiangen, Beijing, China). cDNA synthesis was conducted using a cDNA reverse transcription kit (KR116-02, Tiangen, Beijing, China). Realtime-PCR was performed using a SYBR Green PCR Master Mix (BL698A, Biosharp, Anhui, China). Each sample was measured in triplicate, and we assessed expression differences using the 2^–ΔΔCT^ method (Livak and Schmittgen, 2001). The primer sequences are shown in Table 1.

**TABLE 1:** Sequences of primer pairs used for Realtime-PCR.

Gene	Primer	5′-3′ Sequence
BDNF	F	CCGGTATCCAAAGGCCAACT
	R	CTGCAGCCTTCCTTGGTGTA
NR1	F	ATCGCCTACAAGCGACACAA
	R	GGATGGTACTGCTGCAGGTT
NR2A	F	CATTGGGAGCGGGTACATCTT
	R	CGTCACCAACAAACTGGAGC
NR2B	F	GGGTCACGCAAAACCCTTTC
	R	GGCTGACACCACTGGCTTAT
Caspases-3	F	GTCATCTCGCTCTGGTACGG
	R	CACACACACAAAGCTGCTCC
Bax	F	CTCAAGGCCCTGTGCACTAA
	R	CACGGAGGAAGTCCAGTGTC
Bcl-2	F	TCTTTGAGTTCGGTGGGGTC
	R	AGTTCCACAAAGGCATCCCAG
β-actin	F	TTGCCGACAGGATGCAGAAG
	R	AGGTGGACAGCGAGGCCAGGAT

F, forward; R, reverse.

### 2.13 PCR array

The total RNA was extracted according to the previous steps. Utilizing the PCR Array (WC-MIRNA0023-H, WcGENE, Shanghai, China) and following the manufacturer’s instructions, the polymerase chain reaction was conducted using the BIO-RAD detection system. The relative abundance of the gene was calculated using the 2^–ΔΔCT^ formula, with U6 serving as the reference gene.

### 2.14 Oxygen–glucose deprivation (OGD)

Mouse hippocampal neurons (HT22, Pricella CL-0697) are used to simulate neural cells in the brain, and the treatment of OGD was performed to mimic cerebral ischemia reperfusion (I/R). Morphology and growth status of HT22 were identified under a phase-contrast microscope. Procedures for OGD induction were as follows. HT22 were cultured in a glucose-free medium under conditions of 95% N_2_ and 5% CO_2_ at 37°C for 4 h to induce OGD.

### 2.15 Cell viability

Cell viability was detected by CCK-8. Briefly, HT22 cells were seeded in 96-well plates with a density of 1 × 10^5^ cells per well. The cells were treated with OGD for 4 h, and then 10 μL of CCK-8 solution (CA1210, Solarbio, Beijing, China) was added to each well for 4 h at 37°C. The optical density at 450 nm was measured using microplate reader to detect cell viability.

### 2.16 Scratch healing assay

Perform a scratch healing assay to assess cell migration. Researchers will observe cell migration under a phase contrast microscope every 24 h and capture images. Use Image-J software to analyze the migration distance of cells.

### 2.17 Western-blot

We prepared total cell extracts using a previously reported protocol (Wu et al., 2017). Primary and secondary antibodies used for immunoblotting are reported in Table 2. Immunoreactive protein visualization was performed using an ECL detection system (Bio-RAD, CA, United States), and Image J analysis software.

**TABLE 2:** Antibodies used in the study.

Antigen	Antibody	Cat #
Cleaved caspase-3	Cleaved Caspase-3 (Asp175) (5A1E)	#9664 - CST
bcl-2	Bcl-2 (D17C4)	#3498 - CST
Bax	Bax antibody	#2772 - CST
NR2A	NMDAR2A/GRIN2A	#28525-1-AP - Proteintech
NR2B	NMDAR2B/GRIN2B	#21920-1-AP - Proteintech
BDNF	BDNF antibody	#66292-1-Ig - Proteintech
β-actin	β-Actin (8H10D10)	#3700-CST

### 2.18 Statistical analysis

Data processing and analysis were conducted using GraphPad Prism 7.0 software (GraphPad Software, San Diego, CA, United States). Results were presented as mean ± standard deviation. Statistical significance was evaluated using one-way analysis of variance (ANOVA), supplemented by Bonferroni *post hoc* tests for multiple comparisons. Two-tailed student′s t was performed for pairwise comparisons. *P* < 0.05 was considered statistically significant.

## 3 Results

### 3.1 Characterization of HUC-MSCs and HUC-MSCs-EVs

**FIGURE 1:**
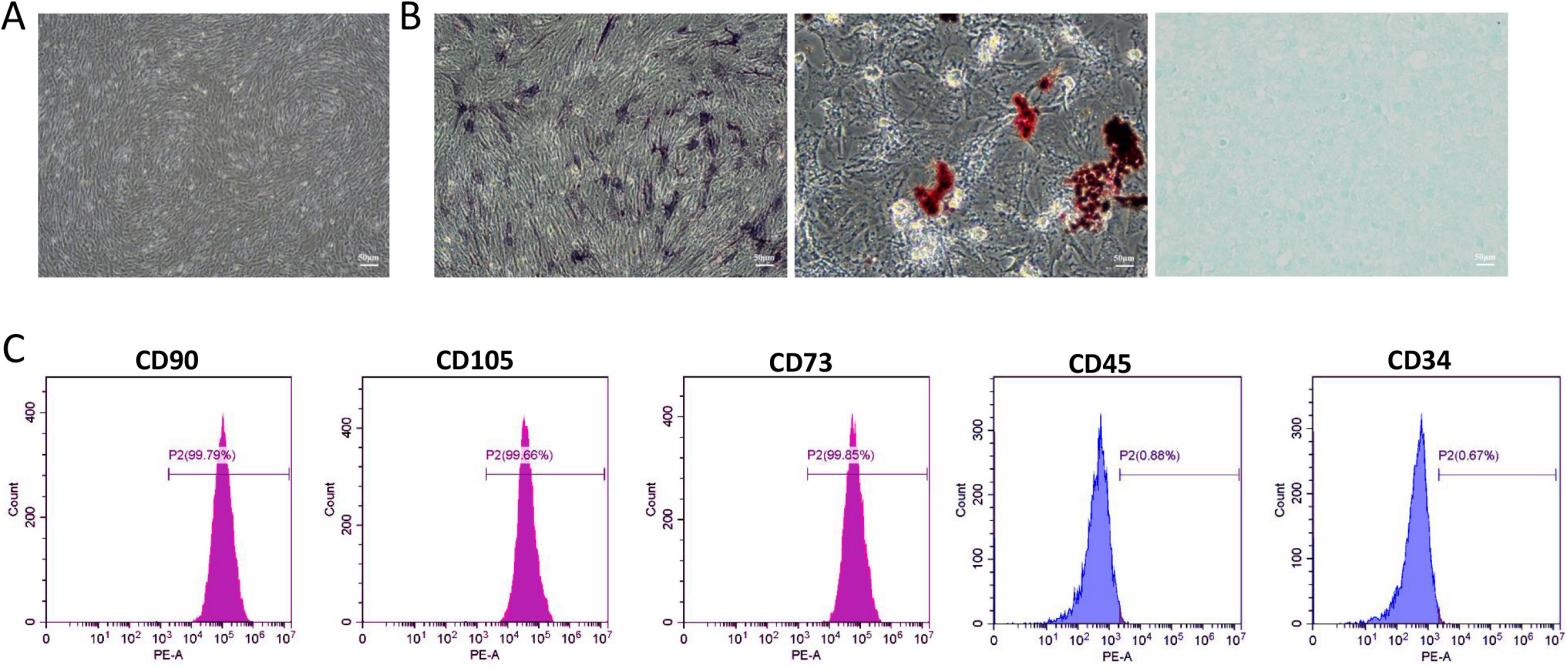
Identification of Human umbilical cord mesenchymal stem cells (HUC-MSCs). **(A)** The passage culture of HUC-MSCs exhibits a uniform spiral arrangement. **(B)** ALP staining indicated that HUC-MSCs differentiate toward the osteogenic lineage; Oil Red O staining indicated adipogenic differentiation; Alcian staining indicated chondrogenic differentiation. **(C)** Flow cytometry analysis of cell surface markers revealed representative histograms for HUC-MSCs. Scale bars: 50 μm in **(A and B)**.

Human umbilical cord mesenchymal stem cells (HUC-MSCs) formed a uniform vortex pattern after passage culture (Figure 1A). Following a triple-lineage differentiation experiment, calcium nodules and intracellular lipid droplets appeared, which were stained with ALP, Oil Red O, and Alcian, respectively (Figure 1B). This indicates that HUC-MSCs have the potential to differentiate into osteogenic, adipogenic, and chondrogenic cells. We performed flow cytometry analysis to determine the phenotype of HUC-MSCs based on their surface markers. The results showed that the HUC-MSCs specific markers CD90 (99.79%), CD105 (99.66%), and CD73 (99.85%) exhibited strong positive reactions. In contrast, hematopoietic cell-specific markers CD45 (0.88%) and CD34 (0.67%) showed negative reactions (Figure 1C). Realtime-PCR was used to detect the mRNA expression levels of NR2A and NR2B in HUC-MSCs treated with different concentrations of memantine. At 200 μM, the expression of NR1, NR2A, and NR2B in HUC-MSCs was significantly inhibited (Supplementary Figure 2). Furthermore, results from the CCK8 assay showed that the cell proliferation ability of HUC-MSCs gradually decreased after treatment with varying concentrations of memantine (Supplementary Figure 3).

**FIGURE 2:**
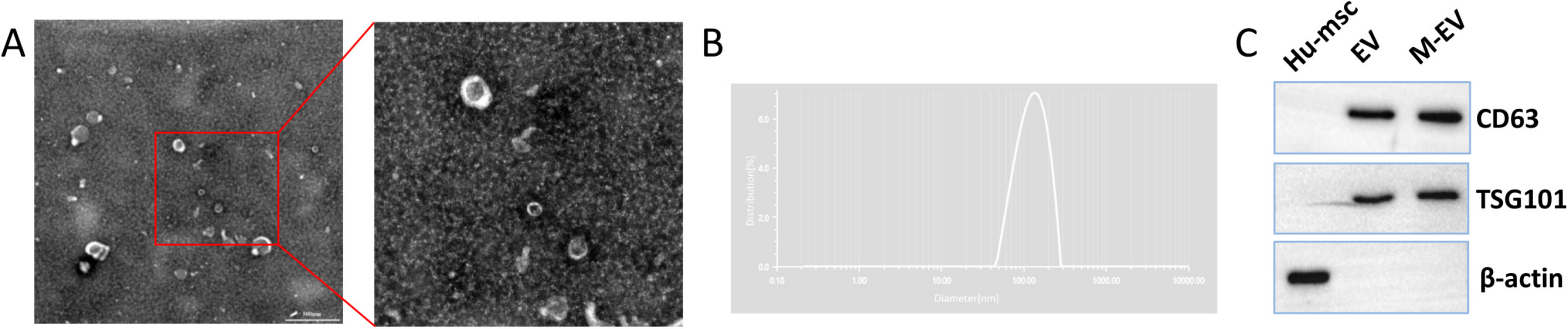
Identification of HUC-MSCs-derived EVs. **(A)** Under TEM, the M-EVs appear cup-shaped. **(B)** Use NTA to analyze the particle size distribution of M-EV. **(C)** Western-blot of extracellular membrane markers CD63 and TSG101. Scale bars: 500 nm in **(A)**.

As shown in Figure 2A, the pre-processed M-EV exhibits a typical spherical or cup-shaped morphology, consistent with the characteristics of EVs (Figure 2A). NTA showed a particle size distribution of 114.61 nm for the pre-processed M-EV, with a peak of approximately 132.48 nm (Figure 2B). In addition, the isolated EVs expressed CD63 and TSG101, which are markers for EVs, but not the non-extracellular marker protein β-actin (Figure 2C). These features indicate that the pre-processed HUC-MSCs-derived particles are EVs.

### 3.2 Preconditioned-EVs attenuated brain damage volume after stroke

**FIGURE 3:**
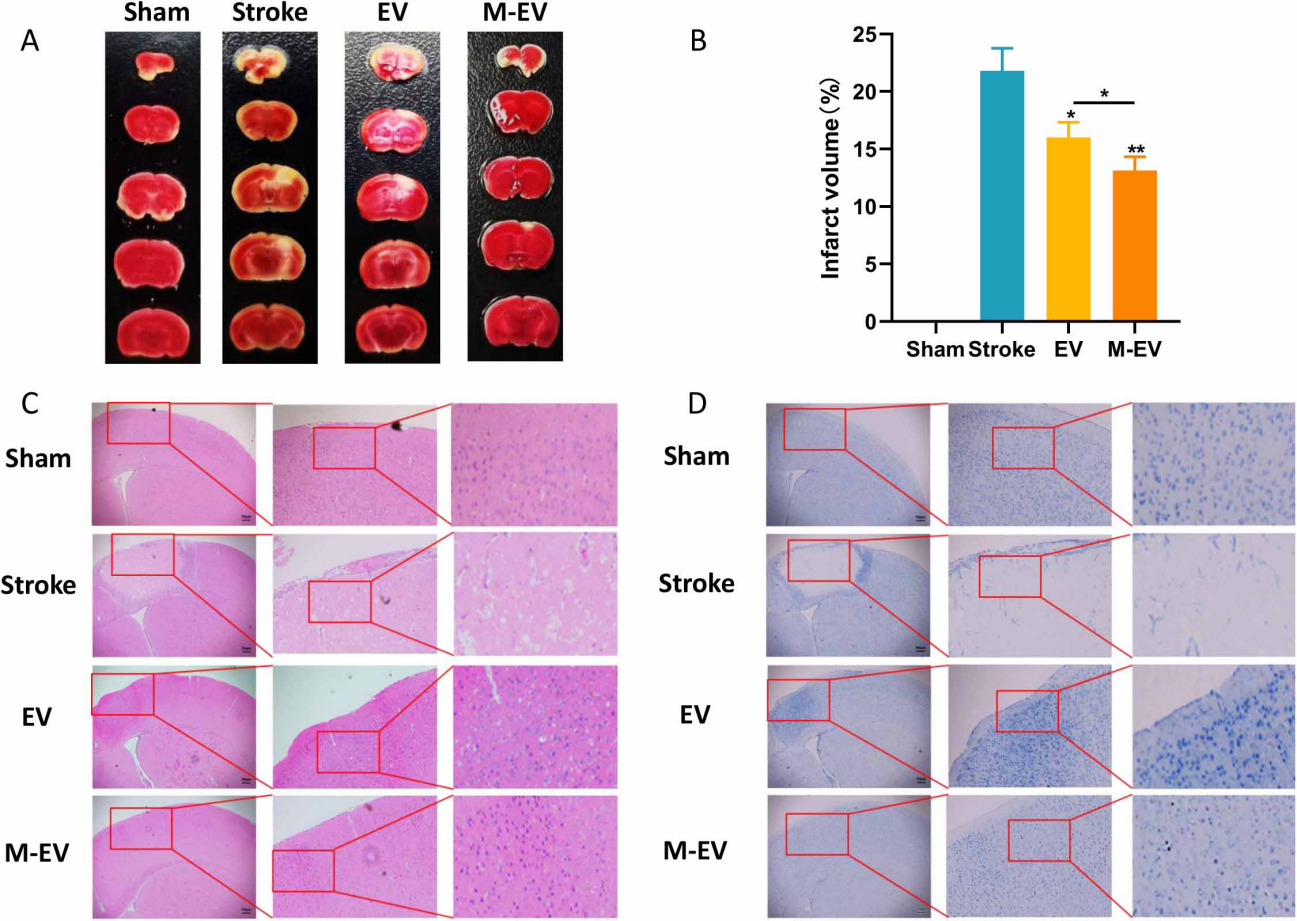
NMDAR inhibitor-preconditioned EVs reduced brain damage volume and neural apoptosis after stroke. **(A)** Representative images of TTC staining. **(B)** Quantitative analysis of cerebral infarction volumes. **(C)** Representative images of HE staining. **(D)** Representative images of Nissl staining. All data were expressed as mean ± SD; *P < 0.05, **P < 0.01. *n* = 3. Scale bars: 50 μm in **(C and D)**.

Triphenyltetrazolium chloride (TTC) staining showed that the normal brain tissue slices of mice with stroke were bright red, while the affected side was light red or white. The volume of cerebral infarction in the stroke group mice was found to be greater than 20%. Injecting EVs after a local cerebral infarction reduces the volume of the infarction. Compared with the EV group, the M-EV group showed a significant reduction in cerebral infarction volume (Figures 3A, B). HE staining showed that the cells in the damaged area of the stroke group were tightly arranged and disappeared, and the peripheral nerve nuclei were wrinkled and deformed (Figure 3C). Nissl staining also showed that M-EV showed a higher density and wider dispersion of Nissl bodies, with a larger area than the Stroke and EV groups (Figure 3D). Injecting M-EV after cerebral ischemia can aid in the repair of nerve fibers and neurons.

### 3.3 Preconditioned-EVs improved neurological function after stroke

**FIGURE 4:**
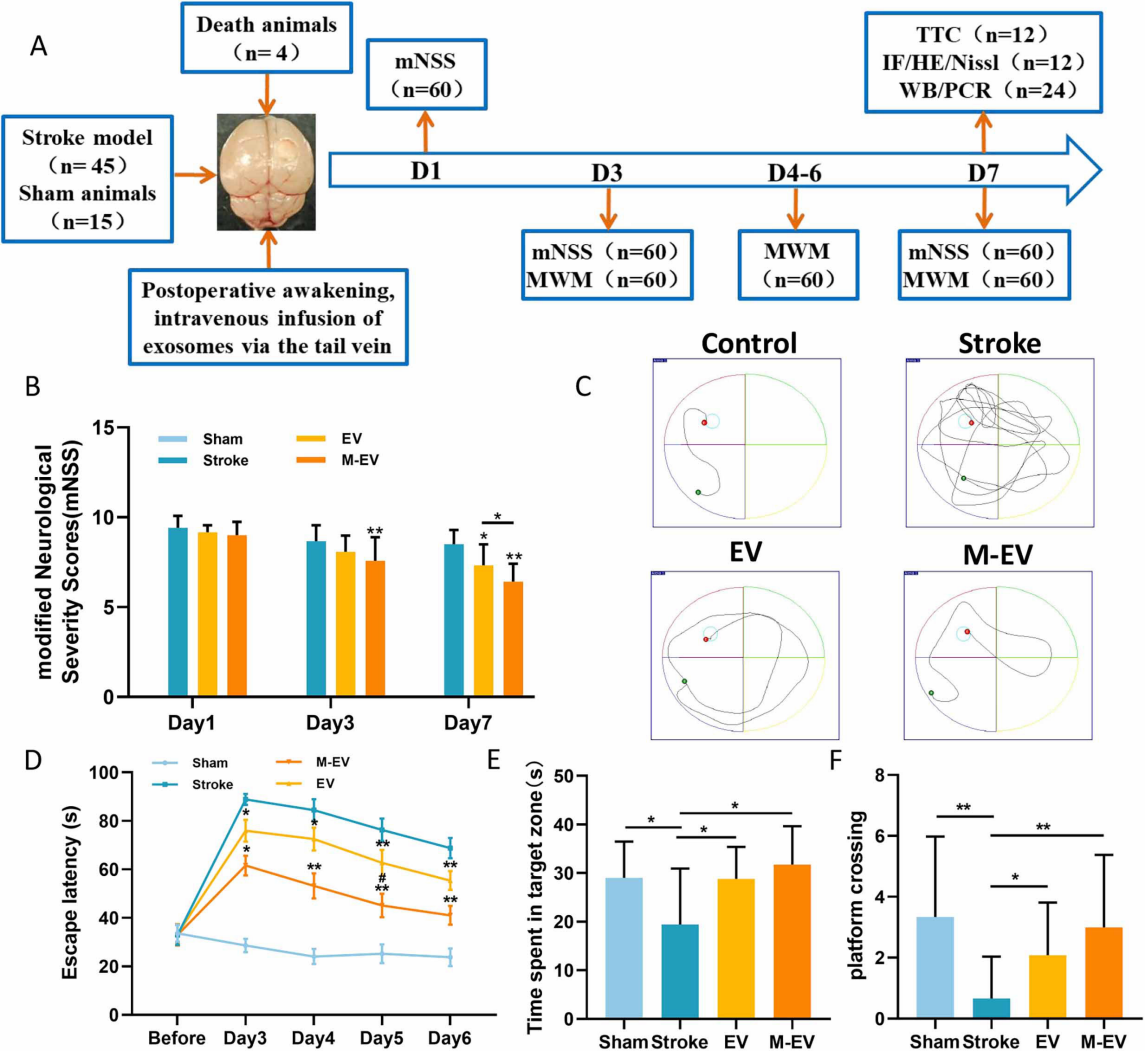
NMDAR inhibitor-preconditioned EVs improved spatial cognition and sensorimotor integration in stroke models. **(A)** Experimental program timeline: The figure displays the allocation of all groups and the number of animals involved. **(B)** mNSS scores of mice on days 1, 3, and 7 post-stroke. **(C)** Representative images of the swimming routes of animals in each group. **(D)** Animal incubation period. **(E)** The duration of stay for each group of animals in the target quadrant. **(F)** The number of times each group of animals crosses the platform. All data were expressed as mean ± SD; *P < 0.05, **P < 0.01; ^#^*P* < 0.05. *n* = 12.

We evaluated whether pre-adapted EVs offer an advantage in improving cognitive impairment caused by injury using the mNSS and Morris water maze experiments (Figure 4A). Serial neurological assessments using the mNSS were performed on post-PTI days 1, 3, and 7 in murine models. The Stroke group had the highest mNSS scores, which declined over time. Results revealed significant functional recovery in EV/M-EV cohorts compared to stroke controls, with M-EV demonstrating superior neurobehavioral restoration (Figure 4B).

The water maze results indicated that the spatial memory ability of mice with stroke improved after EVs treatment, as evidenced by the shortening of the search path (Figure 4C). The Stroke group exhibited significant cognitive deficits compared to the Sham group. Compared to the Stroke group, the EV group demonstrated shorter latency, longer dwell time in the target quadrant, increased frequency of platform crossing, and enhanced cognitive function (Figure 4D). Furthermore, the M-EV group, which received M-EV, showed enhanced cognitive recovery in mice with local cerebral infarction compared to the EV group (Figure 4D). It is noteworthy that the M-EV group had a significant increase in dwell time and platform crossing frequency in the target quadrant compared to the EV group (Figures 4E, F). These findings suggest that M-EV have an additional restorative effect on cognitive impairment caused by stroke.

### 3.4 Preconditioned-EVs promoted cerebrovascular remodeling after stroke

**FIGURE 5:**
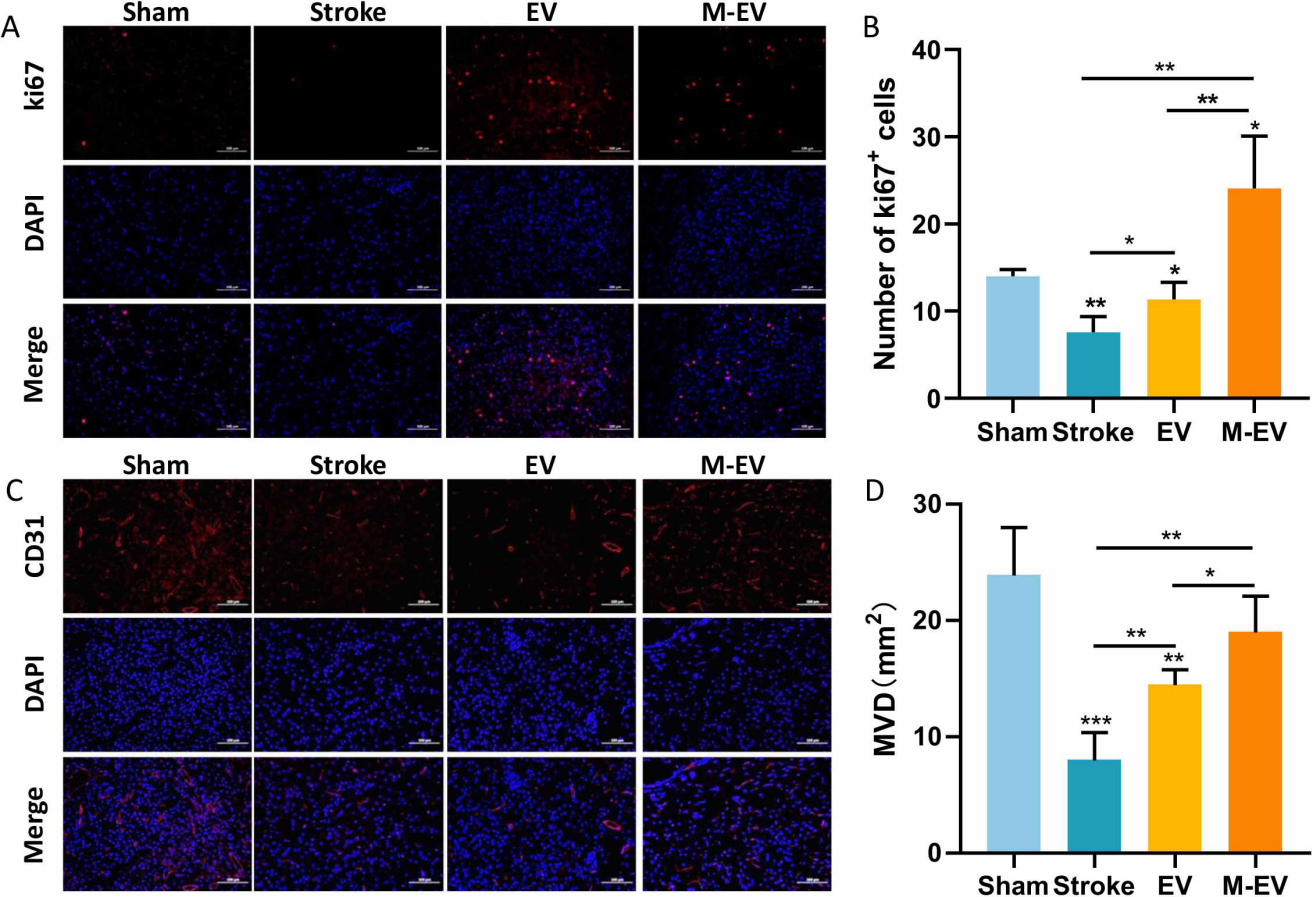
NMDAR inhibitor-preconditioned EVs enhance post-stroke cerebrovascular reconstruction. **(A)** Typical images of immunostaining for CD31 in peri-infarct blood vessels of mice. **(B)** Comparative microvessel density (MVD) profiling via CD31 Immunofluorescence quantification. **(C)** Typical images of Ki67 immunostaining for cellular proliferation around the infarction in mice. **(D)** Quantitative analysis of Ki67-positive proliferating cells in each group. All data were expressed as mean ± SD; *P < 0.05, **P < 0.01, ***P < 0.001. *n* = 3. Scale bars: 100 μm in **(A and C)**.

Previous studies have shown that M-EV therapy can significantly reduce the volume of cerebral infarction and improve neurological damage caused by stroke. Consequently, we further explored the mechanism of M-EV therapy for stroke. We observed cerebral vascular remodeling 7 days after local cerebral infarction induced by light clot, using immunofluorescence staining with the cell proliferation marker Ki67 and the endothelial cell marker CD31 (Figures 5A–D). Compared with the Stroke group, both the EV group and M-EV group exhibited a significant increase in the number of Ki67-positive cells, indicating enhanced cell proliferation (Figures 5A, B). In addition, quantitative morphometry revealed superior cerebrovascular reorganization in M-EV-treated subjects versus conventional EV groups (Figures 5C, D).

### 3.5 Preconditioned-EVs promoted neuroprotection after stroke

**FIGURE 6:**
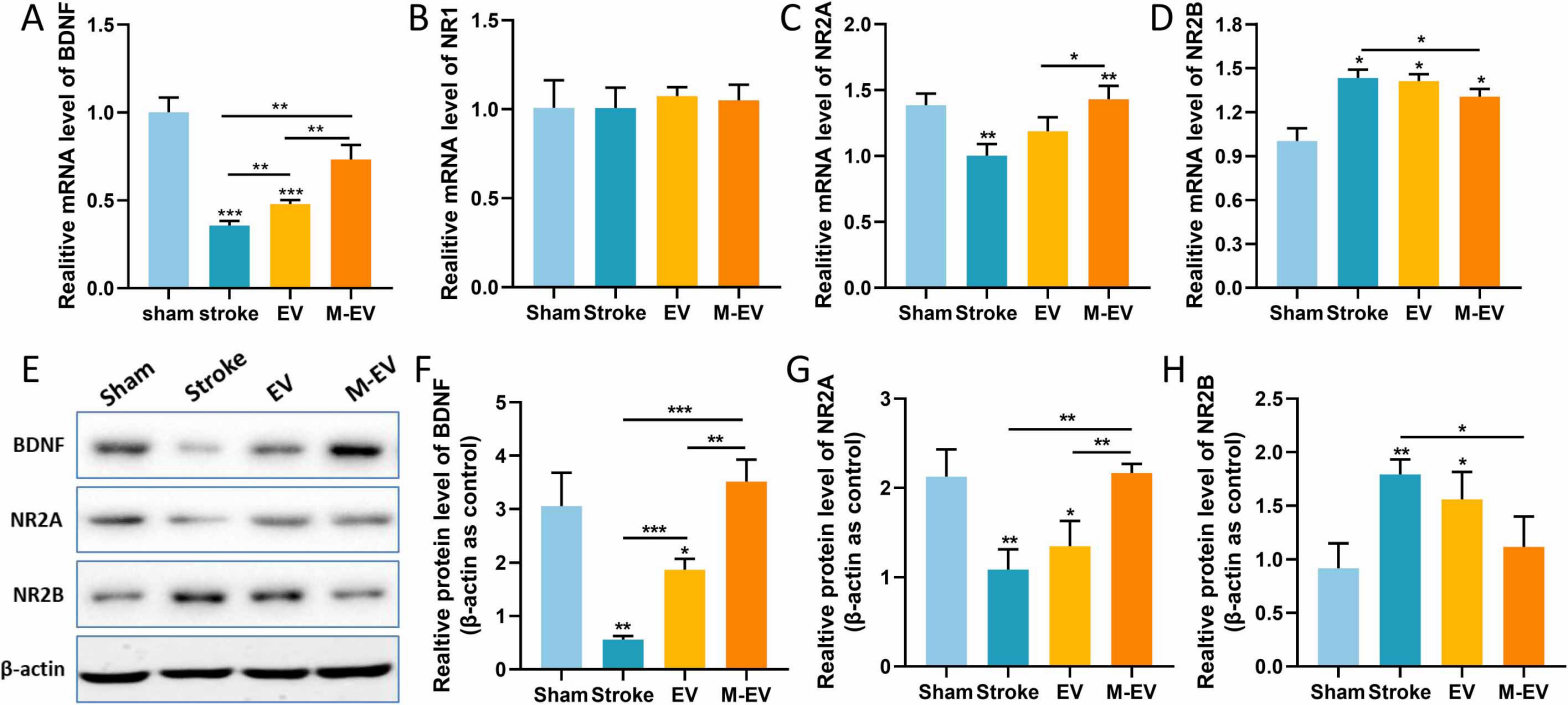
NMDAR inhibitor-preconditioned EVs inhibited the production of glutamate excitotoxicity after stroke. **(A–D)** Quantitative analysis of BDNF, NR1, NR2A, and NR2B expression levels in brain tissue using Realtime-PCR. **(E)** Western blotting was used to detect the protein expression levels of BDNF, NR2A, and NR2B in brain tissue. **(F–H)** Quantitative analysis of **(E)**. All data were expressed as mean ± SD; *P < 0.05, **P < 0.01, ***P < 0.001. *n* = 3.

To investigate whether functional recovery induced by HUC-MSCs-EVs can improve outcomes in stroke, we employed Realtime-PCR and Western-blot analysis. We found that after the injection of EVs, the expression of Brain-derived Neurotrophic Factor (BDNF), which is crucial for the survival of brain neurons, increased in the damaged hemisphere of the mouse brain (Figures 6A, E, F). In stroke mice injected with M-EV, BDNF expression was significantly higher, promoting the recovery of damaged neurons.

The NR1, NR2A, and NR2B subunits of the NMDAR are the main subtypes expressed in the forebrain of mammals and are associated with stroke (Monyer et al., 1994). We therefore assessed the mRNA and protein expression levels of these subunits in the damaged brain tissue. Quantitative PCR analysis demonstrated comparable NR1 mRNA expression revealed no variation among treatment arms (*p* > 0.05). (Figure 6B). Following ischemic stroke in mice, the expression level of NR2A in the Stroke group decreased, while that of NR2B increased. After the injection of EVs, NR2A exhibited significant transcriptional up-regulation concomitant with NR2B down-regulation. In stroke mice injected with M-EV, the expression of NR2A was significantly higher, and that of NR2B was significantly lower compared to the Stroke group (Figures 6C–E, G, H).

### 3.6 Screening of EVs differentially expressed miRNAs and analysis of target genes

**FIGURE 7:**
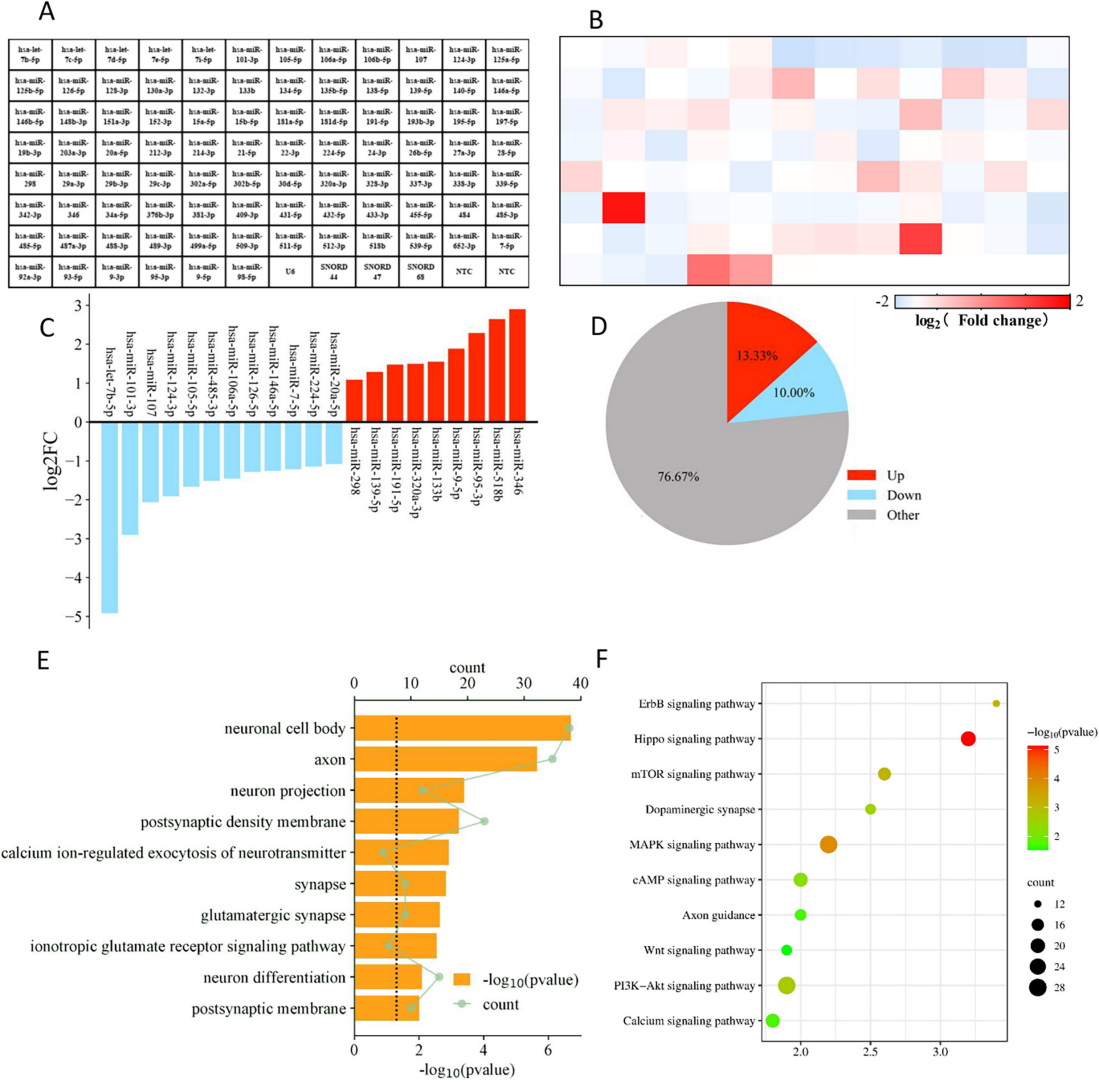
Enrichment profiling delineates NMDAR-inhibitor-preconditioning-induced pathway rewiring via miRNA-mRNA regulatory networks. **(A)** Flat layout of polymerase chain reaction chip. **(B)** The heatmap displays the differential gene expression between the M-EV group and the EV group. **(C)** Main differentially regulated micRNAs. **(D)** The pie chart displays the number of differentially regulated genes. **(E)** The top 10 GO functions of predicted targets in neural differentiation. **(F)** The top 10 KEGG Pathway enrichment analysis of differential expression genes in neural differentiation.

We performed PCR Array detection on EV and M-EV secreted by HUC-MSCs, analyzed microRNAs associated with neurological development and disease, and plotted heatmaps (Figures 7A, B). Comparative miRNA profiling identified 21 differentially expressed miRNAs between EV and M-EV groups, with 42.9% (9/21) demonstrating significant upregulation (fold change ≥ 2.0, *p* < 0.05), including hsa-miR-298, hsa-miR-139-5p, hsa-miR-191-5p, hsa-miR-320a-3p, hsa-miR-133b, hsa-miR-9-5p, hsa-miR-95-3p, hsa-miR-518b, and hsa-miR-346 (Figure 7C). And 12 miRNAs were down-regulated, including hsa-let-7b-5p, hsa-miR-101-3p, hsa-miR-107, hsa-miR-124-3p, hsa-miR-105-5p, hsa-miR-485-3p, hsa-miR-106a-5p, hsa-miR-126-5p, hsa-miR-146a-5p, hsa-miR-7-5p, hsa-miR-224-5p, and hsa-miR-20a-5p (Figure 7D).

We conducted GO analysis on target genes and found that biological process (BP) genes are primarily involved in biological regulation, cellular processes, and developmental processes. Cellular component (CC) genes are mainly associated with the nucleus, cell membrane, and organelles, while molecular function (MF) genes are predominantly involved in binding and catalytic activities (Figure 7E). These findings suggest that EVs secreted by HUC-MSCs may play a critical role in cellular communication and coordination, influencing both the physiological and pathological states of cells. KEGG pathway analysis revealed that the target genes are primarily enriched in signal transduction pathways, including those involved in regulating axonal regeneration, such as the PI3K-Akt signaling pathway, the MAPK signaling pathway, and the mTOR signaling pathway (Wang and Wang, 2020). The KEGG analysis suggests that M-EV may regulate cell proliferation and apoptosis, as well as neurotransmitter release at synapses, potentially improving neurological damage caused by stroke (Figure 7F).

### 3.7 Preconditioned-EVs improved HT22 cell viability, migration and reduced cell apoptosis

**FIGURE 8:**
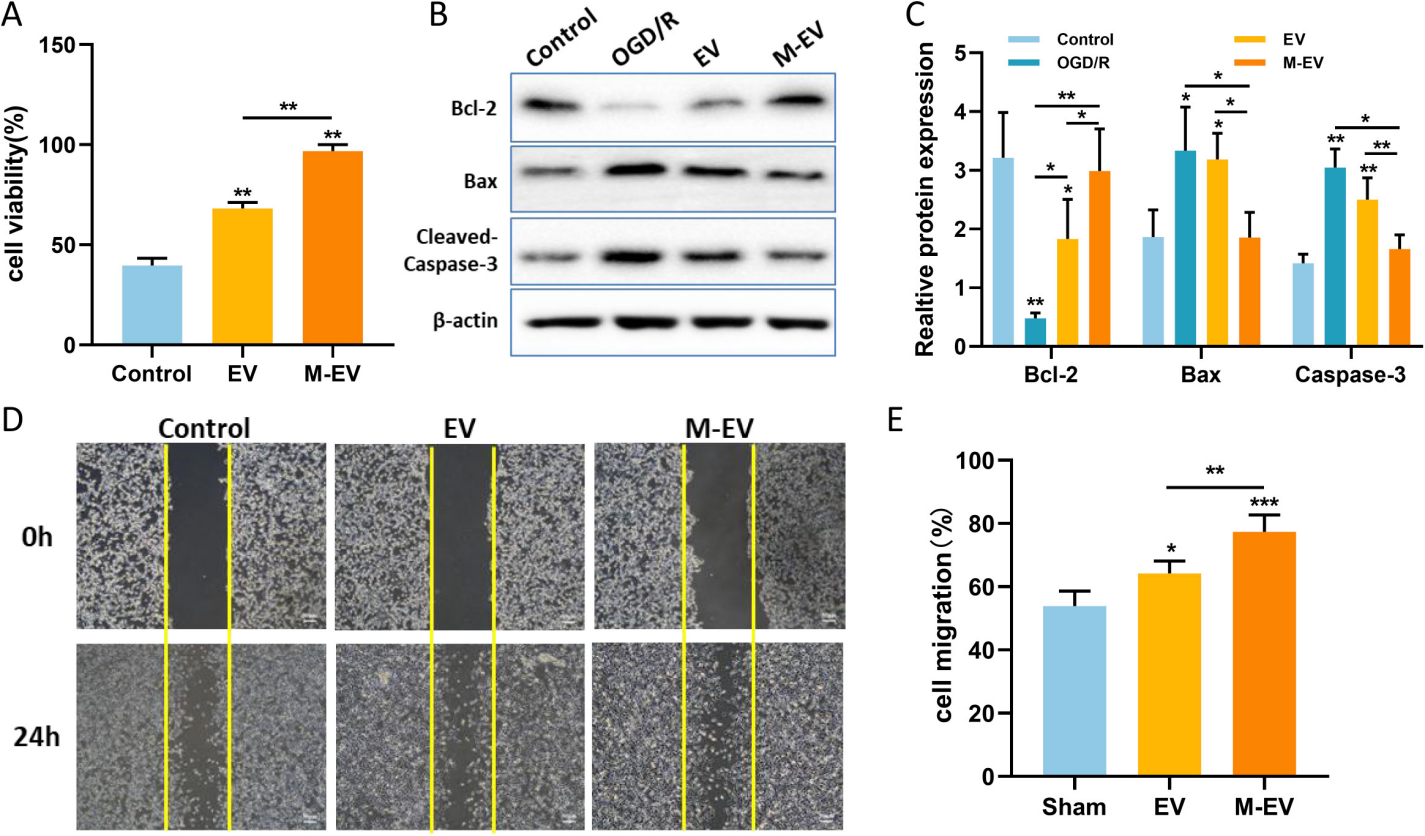
NMDAR inhibitor-preconditioned EVs promoted proliferation, migration and inhibited apoptosis of HT22 cells after OGD. **(A)** CCK8 detection of proliferation of HT22 cells. **(B)** Western blotting was used to detect the expression levels of Bcl-2, Bax, and Cleaved-Caspase-3 in HT22 cells after OGD. **(C)** Quantitative analysis of **(B)**. **(D)** Typical images documenting wound healing progression in treatment groups at 0 and 24 h post-scratch. **(E)** Quantitative analysis of **(D)**. All data were expressed as mean ± SD; *P < 0.05, **P < 0.01, ***P < 0.001. *n* = 3. Scale bars: 50 μm in **(D)**.

To further validate the contribution of M-EV to neuronal injury repair, we used the CCK8 assay to detect the proliferation of neuronal cells. After adding EV and M-EV, the proliferation ability of HT22 cells was enhanced, with the M-EV group exhibiting a higher proliferation ability than the EV group (Figure 8A). The western-blot results (Figure 8B) showed that OGD induced an increase in Bax and Cleaved-Caspase-3 expression, while Bcl-2 expression decreased. After adding EVs, the expression of Bax and Cleaved-Caspase-3 was significantly reduced, and the expression of Bcl-2 was up-regulated (Figure 8C).

The scratch wound healing assay is used to evaluate cell migration ability. The initial scratch width was consistent among the control group, the EV group, and the M-EV group. The migration rate of the EV group and M-EV group at 24 h was higher than that of the control group (Figure 8D), and the M-EV group demonstrated a significantly greater scratch healing area compared to the EV group (Figure 8E).

## 4 Discussion

Stroke is the second leading cause of death globally. In clinical practice, a characteristic of this disease is the sudden onset of neurological dysfunction (Hilkens et al., 2024). Ischemic stroke is the most common type of stroke worldwide, accounting for approximately 85% of all stroke cases, and results in about 6.4 million deaths annually (Avan et al., 2019). In the context of stroke, the application of EVs derived from MSCs has been shown to significantly bolster the recuperation of fine motor skills, enhance spatial learning and memory capabilities, diminish the severity of neurological symptoms, and curtail the extent of infarcted tissue (Han et al., 2019; Guy and Offen, 2020). EVs derived from MSCs could represent an optimal therapeutic alternative (Keshtkar et al., 2018). EVs have demonstrated the capacity to mirror the reparative functions of their parent cells, contributing to the restoration of various tissues and organs (Wu et al., 2019; Yao et al., 2022). The NMDAR antagonist memantine is considered a potential treatment for ischemic stroke, with studies indicating that low concentrations of memantine improve post-stroke behavioral outcomes. However, high concentrations of memantine significantly exacerbate the damage (Trotman et al., 2015). The failure of memantine in stroke clinical trials may be attributed to significant side effects, a narrow therapeutic window, lack of vascular protection in restoring local blood supply, and the complexity of cellular and molecular injury mechanisms in the human brain (Hoyte et al., 2004; Muir, 2006; Ikonomidou and Turski, 2002). Despite these obstacles in the development of stroke therapies, ongoing research confirms that NMDAR-mediated excitotoxicity is a primary mechanism of cell death (Choi, 2020). Therefore, it can be hypothesized that M-EV can play a crucial role in neural injury repair.

In recent years, the long-standing belief that brain cells are incapable of regeneration has been upended by the discovery of new neurons in the hippocampus and the migration of stem cells in animal models (Hoang et al., 2022; Dumitru et al., 2025). This has opened the door to the possibility of using exogenous stem cells to augment or replenish the brain’s stem cell pool, offering a hope for the regeneration of neurons in the face of neurological diseases (Carney and Shah, 2011). The procurement of HUC-MSCs is non-invasive, and since umbilical cords are typically discarded as medical waste after birth, there are no ethical concerns associated with their use (Witkowska-Zimny and Wrobel, 2011). HUC-MSCs have demonstrated their potential in regenerative therapies for a range of neurological disorders, including ischemic stroke (Tanaka et al., 2018; Ercelen et al., 2023). Post-stroke, the brain is deprived of oxygen, leading to cell death and an overactive inflammatory response. HUC-MSCs-EVs have been found to not only traverse the blood-brain barrier (BBB) when administered intravenously or intranasally but also to exert beneficial effects on chronic inflammation and promote healthy healing processes, positioning them as a promising treatment for complex brain injuries (Dabrowska et al., 2019; Kharazi and Badalzadeh, 2020; Rahmani et al., 2020). The administration of HUC-MSCs-EVs in stroke models has been shown to provide long-term neuroprotection, enhance neurogenesis and neurovascular remodeling, and improve behavioral and neurological performance in areas such as motor function, coordination, sensorimotor abilities, and spatial learning (Zhang et al., 2019; Zagrean et al., 2018; Xin et al., 2013; Zheng et al., 2021).

Hypoxic and toxin-accumulating microenvironment leads low neonatal cell survival and maturation, while appropriate vascular endothelial remodeling is beneficial for neuronal survival (Ye et al., 2022). Our results indicated that there is a higher quantity of CD31 and Ki67 in the M-EV group compared to the EV group. The neuroprotective role of BDNF is well recognized and plays a pivotal part in therapeutic studies for neurological conditions (Colucci-D’Amato et al., 2020). Boosting the expression of BDNF has the potential to alleviate cell death, stimulate the growth of new neurons and blood vessels, significantly contributing to the neurological rehabilitation of individuals who have suffered a stroke (Liu et al., 2020b; Schäbitz et al., 2007). Our findings suggest that EVs preconditioned with memantine are capable of more effectively reversing the decrease in BDNF levels induced by cerebral infarction. NMDARs are heterotetramers formed by one GluN1 subunits and two glutamate binding GluN2 subunits (Ge et al., 2020; Wu and Tymianski, 2018). Among these subunits, GluN1, GluN2A and GluN2B are the most abundant in the brain. In the hippocampus of rats, NR2A-containing NMDARs located at synaptic sites are associated with cell survival, whereas NR2B-containing NMDARs located at extrasynaptic sites are linked to cell death (Ge et al., 2020; Martel et al., 2012). Studies have shown that in ischemic brain injury, the activation of GluN2B leads to excitotoxicity and neuronal apoptosis, while the activation of GluN2A results in neuronal survival and neuroprotection against ischemic damage (Liu et al., 2007; Chen et al., 2008). In this study, M-EV reversed glutamate-induced down-regulation of NR2A expression and up-regulated NR2B expression, suggesting a potential mechanism for its neuroprotective effects. Previous studies indicate that NMDAR-mediated PI3K/Akt pathway activation protects neurons against hypoxic and excitotoxic death (Soriano et al., 2006), while pathway inhibition worsens ischemic neuronal death (Niu et al., 2024). However, our findings do not support PI3K/Akt pathway involvement in M-EV’s neuroprotective action. Lv et al. reported that memantine pretreatment improved human umbilical cord endothelial cells (HUVECs) viability and prevented the decrease in microtubule formation induced by OGD/R (Lv et al., 2020). Furthermore, *in vitro* experiments indicate that M-EV can effectively reduce apoptosis in HT22 cells and promote cell proliferation and migration. Therefore, the NMDAR/PI3K/Akt signaling pathway may be a potential mechanism for the neuroprotective effect of EV pre-treated with memantine.

To further confirm the capacity of M-EV to promote neural injury recovery, PCR array analysis was conducted to identify differentially expressed miRNAs associated with neurological development and disease. The up-regulation of miR-139-5p in the preconditioned EVs was found to inhibit the anti-apoptotic effects of the FoxO1/Keap1/Nrf2 pathway, thereby potentially alleviating the impact of ischemic stroke (Yao et al., 2022). Similar studies have corroborated that the overexpression of miR-133b in MSCs leads to a significant enhancement in functional recovery among rats subjected to middle cerebral artery occlusion (MCAO) (Xin et al., 2017). Our PCR array analysis prioritized miR-139-5p and miR-133b as plausible candidates underlying M-EV-mediated neuroprotection; however, their target-specific regulation of apoptotic machinery remains to be functionally interrogated. Furthermore, additional GO and KEGG enrichment analyses have demonstrated that EVs preconditioned with memantine modulate cell proliferation, apoptosis, and neurotransmitter release at synaptic junctions. Concurrently, the target genes are significantly enriched in pathways such as PI3K-Akt, MAPK, and mTOR. Additionally, a peer-reviewed study has validated the association of extracellular miRNAs derived from hypoxia-preconditioned neural stem cells (NSCs) with signaling pathways, including PI3K-Akt, Hippo, MAPK, mTOR, as well as endocytosis (Zhang et al., 2018). Multiple studies have demonstrated that the miRNA composition within EVs fluctuates across various stages of stroke (Jiang et al., 2022). Current research indicates that EVs conditioned with memantine are enriched with miRNAs that facilitate the repair of nerve damage.

## 5 Conclusion

In summary, our findings demonstrate that EVs derived from M-EV selectively alter miRNA loading and enhance therapeutic efficacy. By comprehensively modulating the ischemic brain microenvironment, M-EV significantly improved neurological functional recovery. These results suggest potential clinical applications for managing brain disorders.

## Data Availability

The datasets presented in this study can be found in online repositories. The names of the repository/repositories and accession number(s) can be found in the article/Supplementary material.
