# Supplementary material for: NMDAR inhibitor preconditioned mesenchymal stromal cell-derived extracellular vesicles enhance post-stroke recovery by targeting excitotoxicity and neuronal regeneration
Source: Front Cell Neurosci. 2025 Aug 12;19:1608615. doi: 10.3389/fncel.2025.1608615 (PMC12378720; doi:10.3389/fncel.2025.1608615)
Supplement: Supplementary file 1 [file Data_Sheet_1.pdf]

Supplementary figure

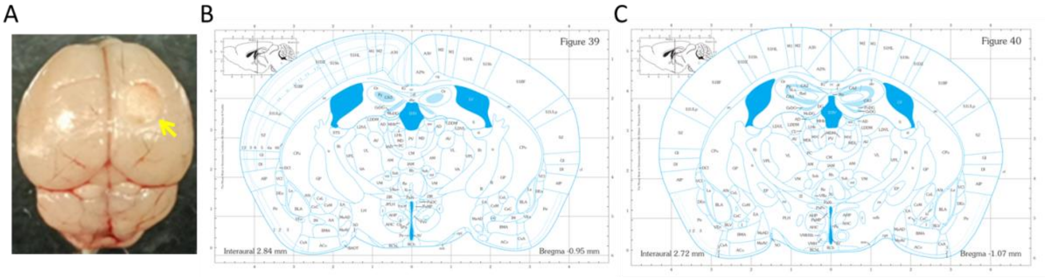

Figure S1. Schematic diagram of stroke injury location. (A) Schematic diagram of the position of the Photothrombotic model. (B-C) Stereoscopic Localization Atlas of Mouse Brain.

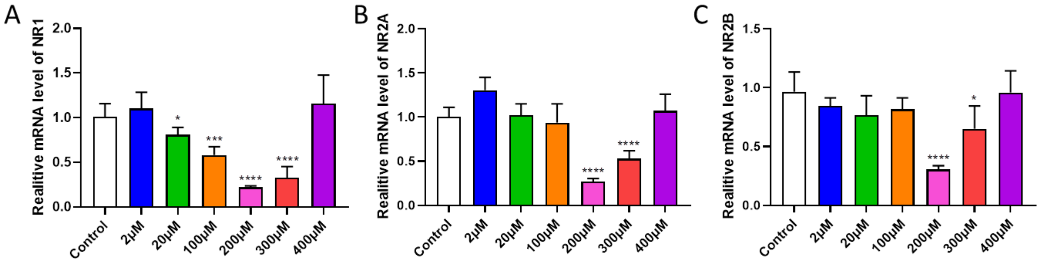

Figure S2. Drug concentration selection. (A-C) Quantitative analysis of NR1, NR2A, and NR2B expression levels in UCMSCs using Realtime-PCR.

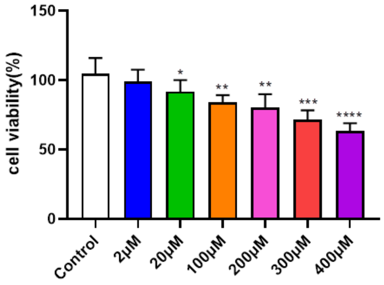

Figure S3. Cell viability detection after drug addition. CCK8 detection of proliferation of UCMSCs cells.
